# The impact of adult neurogenesis on affective functions: of mice and men

**DOI:** 10.1038/s41380-024-02504-w

**Published:** 2024-03-18

**Authors:** Mariana Alonso, Anne-Cécile Petit, Pierre-Marie Lledo

**Affiliations:** 1 Institut Pasteur, Université Paris Cité, CNRS UMR 3571, Perception and Action Unit, F-75015 Paris, France; 2 Pôle Hospitalo-Universitaire Psychiatrie Paris 15, GHU Paris Psychiatry and Neurosciences, Hôpital Sainte-Anne, Paris, France

**Keywords:** Neuroscience, Depression

## Abstract

In most mammals, new neurons are not only produced during embryogenesis but also after birth. Soon after adult neurogenesis was discovered, the influence of recruiting new neurons on cognitive functions, especially on memory, was documented. Likewise, the late process of neuronal production also contributes to affective functions, but this outcome was recognized with more difficulty. This review covers hypes and hopes of discovering the influence of newly-generated neurons on brain circuits devoted to affective functions. If the possibility of integrating new neurons into the adult brain is a commonly accepted faculty in the realm of mammals, the reluctance is strong when it comes to translating this concept to humans. Compiling data suggest now that new neurons are derived not only from stem cells, but also from a population of neuroblasts displaying a protracted maturation and ready to be engaged in adult brain circuits, under specific signals. Here, we discuss the significance of recruiting new neurons in the adult brain circuits, specifically in the context of affective outcomes. We also discuss the fact that adult neurogenesis could be the ultimate cellular process that integrates elements from both the internal and external environment to adjust brain functions. While we must be critical and beware of the unreal promises that Science could generate sometimes, it is important to continue exploring the potential of neural recruitment in adult primates. Reporting adult neurogenesis in humankind contributes to a new vision of humans as mammals whose brain continues to develop throughout life. This peculiar faculty could one day become the target of treatment for mental health, cognitive disorders, and elderly-associated diseases. The vision of an adult brain which never stops integrating new neurons **is** a real game changer for designing new therapeutic interventions to treat mental disorders associated with substantial morbidity, mortality, and social costs.

## Introduction

The discovery that new neurons could be continually generated in the adult mammalian brain has broken a central dogma in Neuroscience and has generated enormous debates among theoreticians, evolutionists, neurobiologists, and clinicians, to name just a few of them [1]. Even though the functional impact of this postnatal neuronal production might vary consistently with species, the ability to continue producing neurons after birth seems to be conserved across evolutionary boundaries, from crustaceans to primates, including humans [2, 3]. However, the degree of this enduring neurogenesis varies according to animal species, a feature that might result from a trade-off between the benefits accrued from recruiting more neurons in a given brain circuit and the disadvantage they generate when integrating an already functioning circuit.

The first observation supporting the existence of adult mammalian neurogenesis came in 1962 when J. Altman discovered it in the cerebral cortex before he made a similar observation in the hippocampus [4]. However, the field of adult neurogenesis has been tugged throughout history by a conceptual resistance (i.e., how long-lasting memory traces could be maintained if the lifespan of neurons is short?) and the lack of appropriate tools to support this provocative concept (i.e., observations of cell divisions in the adult brain was seen initially as proof of gliogenesis rather than neurogenesis). Consequently, it was only in the 1980’s that adult neurogenesis was validated in adult rats by Bayer and colleagues [5, 6]. Together with the studies of Goldman and Nottebohm [7] that extended the same observations in adult brain birds, the scientific community started to renew interest in a phenomenon already known twenty years earlier. Since then, these initial observations have been expanded over time to most mammals including humans when Eriksson and colleagues, in ref. [8], were able to obtain postmortem brain tissue from patients who had been treated with BrdU (5-bromo-3′-deoxyuridine, a thymidine analogue that labels DNA) as a diagnostic biomarker. For the first time, they reported compelling evidence of BrdU labeling in neurons (i.e. NeuN+ cells) in the human dentate gyrus [8]. We know now that two brain regions—the olfactory bulb and the hippocampus—incorporate new neurons throughout life in most mammalian species [9]. In these regions, the cell-level renovation is not static or merely restorative, but it constitutes an adaptive response to challenges imposed by an animal’s environment and/or internal states. A huge amount of work has been performed to understand the cellular and molecular mechanisms controlling adult neurogenesis particularly in the rodent brain (for reviews see refs. [10, 11]). More recent studies have reported the presence of a constitutive adult neurogenesis in other brain regions including the striatum, amygdala, olfactory cortex [12], and the hypothalamus [13] (see Box 1). We focus the present review on the numerous evidence supporting adult-neurogenic potential of the mammalian brain and the links associating this process with psychiatric disorders.

Box 1 Adult neurogenesis comes in the plural In rodents, adult neurogenesis has been extensively studied using the subventricular zone (SVZ) -olfactory bulb system. There, astrocytes located in the adult SVZ act as slow-dividing neural stem cells capable of generating a progeny of neuroblast precursors. These neuroblasts proceed towards the olfactory bulb along the rostral migratory stream. Once in the bulb, new cells mature principally into local inhibitory neurons, but few of them give rise to excitatory neurons [180]. The subgranular zone (SGZ) of the hippocampus is another neurogenic niche in the adult brain. It lines the hilar side of the granule cell layer of the dentate gyrus. There, astrocytes give rise to intermediate progenitors, producing about 9,000 new neurons per day in young adult rats [181]. These progenitors mature locally into granule neurons in the dentate gyrus, sending axonal projections into the CA3 and CA2 regions and dendritic arbors into the molecular layer [9]. Some evidence also suggests the presence of constitutive neurogenesis in other brain regions, including the hypothalamus where neurogenesis has the potential to affect metabolism, fat storage and sexual functions [182]. In contrast, the function of adult neurogenesis reported in the striatum, amygdala, substantia nigra, and cortex remain still unclear [183]. Searching for the source of new neurons in the adult brain, some evidence indicates that newly-generated neurons in the striatum or amygdala are derived from the SVZ, while in the hypothalamus, the proliferation of progenitor cells occurs in the ependymal cells lining the third ventricle. The degree of adult neurogenesis depends particularly on environmental exposures, as reported in the hippocampus, the olfactory bulb circuit [184], and in other brain regions [183]. The poor breeding and housing conditions commonly used in laboratories could be responsible for the low level of adult neurogenesis usually seen in captive animals. Raising animals in more natural environments in combination with next-generation sequencing technologies, are required to fully appreciate the precise level of adult neurogenesis and its functional impact.

## Adult neurogenesis in mammals

**Fig. 1:**
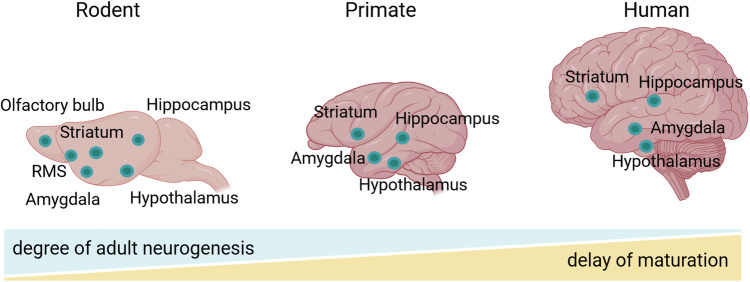
Schematic illustration of the degree of adult neurogenesis and the delay of maturation of new neurons from rodents to humans’ brains. The circles refer to the presence of immature neurons (or de novo production) in the adult brain. The two gradients displayed at the bottom represent the mean strength of each parameter. RMS rostral migratory stream. Created with BioRender.com.

Although systematic studies of hippocampal adult neurogenesis across the whole mammalian phylogenetic scale have not been conducted so far, it is noteworthy that several studies described variations in the degree of adult neurogenesis between species. It is clear today that the maturation rate of new neurons differs across species (Fig. 1). For instance, Kohler and colleagues demonstrate important variations in the maturation rate between the monkeys and rodents [14]. The authors report a slower neural maturation rate in nonhuman primates than in rodents. This property has far-reaching consequences since only the maturing neurons carry unique features by displaying higher excitability and stronger synaptic plasticity [15]. In other words, because the temporal window, in which new neurons are particularly functional, is longer in nonhuman primates than in other species, the functional impact of hippocampal neurogenesis in primates has been so far undervalued. Kohler and colleagues concluded that this protracted period of neuronal maturation confers an important evolutionary advantage by permitting increased cognitive flexibility and discrimination in primates. Below, we review evidence supporting the existence of adult hippocampal neurogenesis in primates.

In the 1980’s, mounting evidence from rodents have quickly convinced the community that the adult brain could integrate new neurons. However, to which extent these findings apply to primates remains controversial. As a consequence, the transfer of results obtained from animal studies to the primates was slowed down. In 1985, using the ^3^H-thymidine labeling approach, Rakic initially reported a lack of adult neurogenesis in non-human primate brains [16] and so the general interest of such studies vanished. However, the possibility of using a non-radioactive cell division marker, BrdU, has provided a new momentum to the field. Combining BrdU with immunohistochemistry became so easy to be used that a new enthusiasm was reborn revealing persistent neurogenesis in adult marmosets [17] and macaques [18–21], though the degree of neurogenesis was found to be 10 times lower than in rodents. More recently, this striking difference in the degree of adult neurogenesis was interpreted as a consequence of a key developmental process. Sorrells and colleagues [22] demonstrated that in monkey, the proliferation of hippocampal neurons sharply decreases during juvenile development while postnatal neurogenesis in rodents remains stable. Thus, adult neurogenesis in the primate hippocampus is considered less robust after the juvenile stage compared to that in rodents and other mammals, because neuronal progenitors exhibit both longer cycles of turnover and slower maturation rates (Fig. 1). This particular kinetic led the authors to conclude that recruitment of young neurons to the primate hippocampus is reminiscent of a long-lasting developmental process that starts during embryogenesis and ends at early stages after birth.

The lack of consensus on adult neurogenesis in non-human primates has had tremendous consequences for neuroscientists since it was considered as a prerequisite to validate its presence in humans. In other words, absence of proof of adult neurogenesis in primates was considered from an evolutive point of view, to be inconsistent with the formation of new neurons in humans. The advent of new technologies, such as single-cell RNA sequencing (RNA-seq), has provided new insights to counteract this objection. Using RNA-seq analysis to reveal molecular diversity and cellular heterogeneity in hippocampus, across the lifespan of macaques, adult neurogenesis was unambiguously characterized [23], making estimable again its study.

## Adult neurogenesis in humans: new evidence on an old debate

The first observations of new neurons generated in the adult human brain were reported in the late 1990s [8] when it became possible to apply a panel of cell division markers on postmortem brain tissue and visualize the results using confocal microscopy (Table 1; see also [3]).

**Table 1:** Principal studies on postnatal neurogenesis in humans.

	Study	Technique	Region	Health status & treatments	Cause of death	Proliferation in adult brain	Immature progenitors in adult brain
No neurological or psychiatric diseases	Eriksson et al. [8]	IHC (BrDU)	HIP	carcinomas tongue, larynx or pharynx No anti-cancer therapy	ns	^a^evidence of proliferation (BrdU+ cells)	
	Sanai et al. [24]	IHC (TuJ1, PSA- NCAM) Neurosphere assay EM	SVZ	tumor, CV, epilepsy Non neurological disease	non-neurological	^a^evidence of SVZ astrocytes proliferation in vivo & in vitro no evidence of RMS	^a^presence of immature progenitors
	Quinones-Hinojosa et al. [25]	IHC (PCNA, Ki67, GFAP)/EM	SVZ-RMS	IO, epilepsy, trauma, glioma, arteriovenous malformation PM no brain pathology	ns	no evidence of RMS	
	Curtis et al. [28]	IHC (PCNA, PSA-NCAM, BrdU), Tunnel+ cells /WB/EM	RMS-OB	carcinomas tongue, larynx, or pharynx	ns		^a^presence of immature progenitors in RMS reaching the OB
	Kam et al. [29]	IHC (PCNA, PSA-NCAM, GFAP, CD133)/EM	SVZ-RMS	non neurological disease	CV, asphyxiation, pulmonal ruptured aneurysm		^a^presence of immature progenitors in RMS reaching the OB
	Sanai et al. [30]	IHC (DCX, PSA-NCAM, GFAP)/EM	SVZ-RMS	non neurological disease but several pathologies vascular malformation, epilepsy, brain tumor, CV, stroke	ns	decline in neurogenesis in early childhood	
	Wang et al. [27]	IHC (DCX, PSA-NCAM, GFAP, β-III tubulin, Ki67, MCM2)	SVZ-RMS-OB	no neuropathology	ns	^a^evidence of proliferation in SVZ	^a^ immature progenitors in SVZ and RMS no neuroblast in the OB
	Bregmann et al. [26]	^14^C in genomic DNA/ IHC (Sox2, HuD)	OB	ns	ns	No evidence of proliferation	
	Martí-Mengual et al. [38]	IHC (DCX, PSA-NCAM, Ki67)	AMY	ctrol, MDD, BD and schizophrenic patients	suicide or ns		^a^presence of immature progenitors
	Spalding et al. [32]	^14^C in genomic DNA	HIP	ns	ns	^a^evidence of proliferation in adults at constant rate	
	Batailler et al. [41]	IHC (DCX, PSA-NCAM, HuC/D)	Hypothalamus	ns	ns		^a^presence of immature progenitors
	Dennis et al. [42]	IHC (Ki67, DCX, PCNA)	LV - HIP- CN	no neurological disease	drowning, Trauma, RF, CV	decline in neurogenesis in early childhood	
	Mathews et al. [43]	mRNA levels (DCX, GFAP, Ki67)/IHC	HIP	no neurological disease	RF, CV	^a^progressive decreases in proliferation with age (mRNA Ki67)	^a^ progressive decreases of immature progenitors with age (mRNA DCX)
	Sorrells et al. [39]	IHC (DCX, PSA-NCAM, Sox2)	AMY	ctrl & ASD	cancer, CV, infection lung disease	no evidence of proliferation in adults	^a^Immature progenitors in PL nuclei persist into old ages
	Boldrini et al. [44]	IHC (Sox2, nestin, DCX, PSA-NCAM, Ki67)	HIP	no neurological or psychiatric diagnoses	ns		^a^ presence of immature progenitors
	Franjic et al [57]	snRNA-seq /IHC	HIP	no neurological disease	atherosclerotic CV, RNF	no immature progenitors detected in adult	
	Wang et al. [23]	snRNA-seq:IHC	HIP	cancer, coma, arteriosclerosis patients	cancer, FR, OF, cancer, infection	^a^evidence of proliferation in adults	^a^ presence of immature progenitors
	Li et al. [40]	IHC (DCX) /WB	TCx -AMY	ctrl	cancers, malformation, CV, infection, MSF, accident		^a^ presence of immature progenitors
Neurological diseases	Curtis et al. [37]	IHC (PCNA)/WB	SEL-CN	ctrl & HD patients	ns	^a^evidence of increased progenitor cell proliferation in the SEL of the HD	^a^presence of immature progenitors
	Boekhoorn et al. [33]	IHC (Ki-67, GFAP, DCX)	HIP	AD patients	cancer, CV, cachexia, RF, VD		^a^presence of immature progenitors No alteration in AD patients
	Liu et al. [34]	IHC (DCX, PSA-NCAM-TuJ1, reelin)/WB	HIP - TCx	ctrl & epileptic patients	ctrl: CV, asphysia,	^a^evidence of proliferation in the normal and epileptic Hipp	^a^presence of immature progenitors in the temporal Cx
	Ernst et al. [36]	^14^C in genomic DNA/ IHC (DCX, PSA-NCAM, idU)/WB	Str	cancer patients (IHC) HD patients	ns	^a^evidence of proliferation Decrease in HD patients	^a^presence of immature progenitors
	Sorrells et al. [22]	IHC (DCX, PSA-NCAM, Sox2) /EM	HIP	ctrl & epileptic patients	trauma, cancer, CV, RF, intraventricular hemorrhage	decline in neurogenesis in early childhood	
	Gatt et al. [35]	IHC (DCX)	HIP	ctrl & Lewy bodies/Parkinson disease dementia patients untreated, SSRI & AChEI-treated	ns		^a^increase immature progenitors in SSRI-treated patients vs Ctrl & untreated patients
	Moreno-Jimenez et al. [49]	IHC (DCX, PSA-NCAM, NeuN, Tuj1)	HIP	ctrl & AD patients	Ctrl: cancer, stroke, sepsis, CV, Leukemia, peritonitis		^a^persistent adult neurogenesis drops sharply in AD patients
	Terreros-Roncal et al. [48]	IHC (DCX, HuC/HuD, PH3 + , Vimentin, PSA-NCAM, Sox2, S100B, Iba1, Nestin)	HIP	ctrl & ALS, HD, α-synucleinopathies, frontal dementia, AD patients	cancer, leukemia, respiratory infection, stroke, RF, peritonitis, CV	^a^changes in proliferation ratio in neurodegenerative diseases	^a^Abnormal adult-born neurons morphological development in neurodegenerative diseases
	Zhou a et al [56]	snRNA-seq/machine learning-based analysis	HIP	ctrl & AD patients	ns	rare proliferating neural progenitors	^a^Presence of immature progenitors with altered gene expression in AD patients
Mood disorders	Boldrini et al. [130]	IHC (Nestin, Ki67, NeuN, GFAP)	HIP	MDD patients, MDD-treated with antidepressant, Ctrl	MDD = suicide & OD ctrl= vehicle accident/CV/peritonitis	^a^evidence of proliferation (increased by antidepressants)	^a^antidepressants increase immature progenitors
	Reif et al. [190]	IHC (Ki67)	HIP	MDD, BD, schizophrenic patients Ctrl (antidepressant, neuroleptic, psychotropic drug)	ns	^a^evidence of proliferation (reduce in schizophrenic patients but not in depression)	
	Lucassen et al. [191]	IHC (MCM2, PH3)	HIP	MDD and bipolar depression patients, treated with antidepressant Ctrl/ non neuropathological alterations	suicide (MDD), CV, cachexia, RF, etc	^a^reduced number of MCM2 + /PH3+ cells in MDD patients	
	Boldrini et al. [131]	IHC (Nestin, Ki67	HIP	MDD untreated, MMD*SSRI & MMD*TCA treated Ctrl: no psychotropic drugs prescription and negative toxicology and psychiatric history	ctrl: CV, peritonitis, MVA MDD: Suicide, OD, RF		^a^increase immature progenitors in MDD*SSRI treated patients
	Epp et al. [132]	IHC (DCX, p21, NeuN)	HIP	MDD w or w/o psychosis	ns		^a^increase immature progenitors in MDD patients (depends on antipsychotics or psychotic symptoms)
Other psychiatric conditions	Bayer et al. [192]	IHC (Musashi, Nestin, Ki67, CR, GFAP, NeuN, TuJ1, DCX)	HIP	heroin addicts no obvious pathologic or traumatic brain disorder	ctrl: CV, Polytrauma, pulmonary Addict: Heroin intoxication		^a^decrease of immature progenitors in drug addicts
	Allen et al. [193]	IHC (Ki67, NeuN)	HIP	schizophrenic patients	ns	^a^reduced proliferation in schizophrenic patients	
	Le Maître et al. [194]	IHC (Ki67, Sox2, DCX)	HIP	ctrl and alcohol abusers	ctrl : CV, Suicide, accident alcohol abuser, suicide, CV, intoxication	^a^reduce proliferation in alcohol abusers	^a^reduce immature progenitors in alcohol abusers

*PM* post-mortem, *IO* intra-operation, *HIP* Hippocampus, *SVZ* subventricular zone, *RMS* rostral migratory stream, *OB* olfactory bulb, *Str* Striatum, *AMY* amygdala, *SEL* subependymal layer, *CN* caudate nucleus, *LV* lateral ventricle, *TCx* temporal cortex, *IHC* immunohistochemistry, *WB* western blot, *EM* electronic microscopy, *CV* cardiovascular, *MSF* multi-system failure, *RF* respiratory failure, *RNF* renal failure, *VD* vascular disease, *OD* overdose, *MVA* motor vehicle accident, *ns* none specified, *ctrl* control, *HD* Huntington disease, *AD* Alzheimer disease, *MDD* Major depressive disorder, *BD* Bipolar disorder, *ASD* autistic spectrum disorder, *ALS* Amyotrophic lateral sclerosis, *SSRI* selective serotonin reuptake inhibitors, *AChEIs* acetyl-cholinesterase inhibitors, *TCA* tricyclic antidepressants.

^a^indicates the studies showing positive evidence for the presence of persistent adult-neurogenesis (including both immature cells and progenitor proliferation).

Since then, the idea of a neuronal formation was extended to the presence of proliferative stem cells in the SVZ [24, 25] even though various techniques failed detecting the presence of new neurons in the olfactory bulb [24, 26, 27]. Consistent with these results the existence of an actual rostral migratory stream in the adult human brain has not been established, or only a few rare dividing cells were found ([27] but see also [28, 29]). The age of the subject seems to matter since the presence of a migratory stream containing dynamic neuroblasts has been observed in children where they integrate the olfactory bulb and the prefrontal cortex, but not during adulthood [30].

Similar controversy occurred on human hippocampal neurogenesis. Although previous studies rule out the presence of new neurons in the hippocampus, using the ^14^C birth dating approach it was reported that humans exhibit similar degree of adult neurogenesis as compared to rodents [31, 32], with a poor decline during aging. Remarkably, this technique has also created a new interest to link neurological diseases with changes in the degree of adult neurogenesis in the case of Alzheimer disease [33], epilepsy [34] and Lewy bodies/Parkinson disease dementia patients [35] (see Table 1; and [3]). Likewise, hippocampal neurogenesis concerns mental health as describe in Table 1. Adult neurogenesis was reported in the striatum but tremendously altered in patients with Huntington’s disease [36, 37]. Importantly, adult-neurogenesis in non-canonical regions (see Box 1), including amygdala [38–40], hypothalamus [41] and temporal cortex [40] was also reported in the human brain.

Despite this new evidence, further inconsistencies still remain in the field. By analyzing proliferation markers typical of young neurons, some studies have ascertained that the degree of postnatal neurogenesis in the dentate gyrus drops sharply in childhood to become absent in adults ([22, 42] but see also [43]). Contrary, Boldrini et al. conclude that human hippocampal neurogenesis persists through life [44]. All these studies were conducted mostly using similar tools based on the detection of the two markers of neuroblasts and immatures neurons, respectively DCX and PSA-NCAM proteins. However, identifying the degree of adult neurogenesis using immunostaining presents numerous technical caveats when performed on postmortem material of human, which might have contributed to the discrepancies [45–47]. Furthermore, the time elapsed between the expression of DCX and PSA-NCAM may vary between species according to the neuronal maturation rate. For instance, Terreros-Roncal et al. [48] demonstrated that PSA-NCAM expression slightly precedes that of DCX in humans. So, the mere expression of cell markers associated with maturating new neurons may be temporally decoupled, explaining why some investigations failed to report co-staining. Finally, the health history of the patients as well as the cause of death, extremely variable between studies (see Table 1), alter the degree of adult neurogenesis.

To clarify some of these issues, Moreno-Jimenez and colleagues have performed a new series of experiments and identified thousands of immature neurons in the hippocampus of healthy subjects, with various ages up to 90 years [49]. The same group then used a combination of antibodies to characterize the development of new neurons to report disrupted hippocampal neurogenesis in patients with Huntington’s disease, fronto-temporal dementia, Parkinson’s disease and amyotrophic lateral sclerosis [48]. These recent studies [44, 48, 50] included the analysis of numerous markers (DCX, PSA-NCAM, Calretinin, Calbindin, Tau, NeuN, Sox2, MAP2, Nestin, Vimentin, S100Beta, HuC, Prox1, etc). This array of proteins and transcription factors include not only markers of immature neurons but also of neural stem cells, progenitors and proliferative cells. Precisely, the same markers that have been used to demonstrate the occurrence of adult neurogenesis in the remaining mammalian species. Although the use of these markers presents several drawbacks, including the fact that commonly used antibodies do not always work on human tissue. Moreover, the group of Llorens-Martin reveals that post-mortem delay, fixation process and antigen retrieval protocols strongly affect the detectability of adult-neurogenesis markers which require a strictly controlled methodology to reconstruct the entire process [3, 51]. The analysis of multiple markers, or of the whole genome, taking consideration of species differences are required to solve the ongoing debate. Whilst this controversy is not yet over [45, 52–54], it is more than likely that the human adult brain remains capable of producing new neurons throughout life.

Given the aforementioned limitations of working with postmortem human samples, the next-generation sequencing technologies emerge with promising potential for alternative evaluation of adult neurogenesis. Single-cell/single-nucleus RNA sequencing (sc/sn-RNA-seq) can be applied for evaluation of the expression profile, cellular diversity, and heterogeneity of tissues at single-cell resolution. Thus, it is useful to characterize various cell types, from stem cells to fully mature neurons [55]. For instance, Zhou and colleagues [56] used sn-RNA-seq in combination with machine learning-based analytic approach to identify immature neurons and quantify their abundance in the human hippocampus. They report the presence of a substantial number of immature neurons in the adult human hippocampus via low frequency de novo generation and prolonged maturation, indicating a major difference compared to other mammalian species (Fig. 1). In the same line, a recent study provides better understanding of hippocampal adult neurogenesis in monkeys and humans by revealing novel markers for distinct cell types [23]. Finally, Franjic and colleagues performed sn-RNA-seq on cells from five subregions of the entorhinal-hippocampal complex from six human donors, as well as on samples from rhesus macaques and pigs [57]. They compared their data with published single-cell sequencing data from mice and observed a homologous trajectory from progenitors to granule cells in pigs and macaques, but not in humans. They found DCX expressed in both mature and immature granule cells, thus demonstrating this marker might not be used as a proxy for adult neurogenesis. In fact, most controversies in the field came from the validation of neurogenesis by means of immunostaining using rodent-derived marker genes. Since immunostaining studies present a significant challenge given the substantial differences between species, next-generation sequencing technologies have offered to the field a revival.

In sum, mounting anatomical, biochemical, and genomic evidence supports the presence of immature neurons in the hippocampus of healthy subjects throughout life. This observation is in sharp contrast with the olfactory bulb where adult neurogenesis seems to be absent, although a new examination taking advantage of more sensitive tools is still missing. How to understand the divergent patterns of adult neurogenesis, different maturation rates, in distinct regions of the mammalian brain from an evolutionary point of view? The well-documented decrease in olfactory abilities that has taken place during the course of evolution, associated with a decrease in the olfactory bulb volume across phylogenetic groups could have been important factors contributing to the extinction of the adult neurogenesis in the primate olfactory bulb [58]. In contrast, the striatum has enlarged in parallel with the cerebral cortex throughout evolution and it is particularly well-developed in higher mammals, including humans. The enlargement of the striatum implies a heavier reliance on movement coordination, cognition, and emotions, possibly requiring a new form of plasticity provided by adult neurogenesis. Raising this possibility is relevant to the “Baldwin effect” in which a culturally invented trait is transformed into an instinctive trait by the means of natural selection repeated throughout generations to increase individual fitness [59]. Debating on the functional relevance of adult neurogenesis seems to be far from over since some studies dismiss it as an evolutionary remnant, given its too low rates, especially in highly cognitively developed species [53].

## Functional impacts of adult neurogenesis on hippocampal circuits

Like many brain regions of the limbic system, the hippocampus is involved in memory, learning, and emotion, in both animals models and humans [60, 61]. It therefore comes as no surprise that increasing the production of new neurons in the hippocampus enhances various cognitive functions, including learning and memory [62], object recognition [63], cognitive representations [64] and forgetting [65] (for review see ref. [66]). Moreover, hippocampal neurogenesis has also been implicated in mood regulation (reviewed in ref. [67]). For instance, studies have shown that reduced neurogenesis in the hippocampus is associated with depression-like behaviors [68], while increasing neurogenesis produces an antidepressant effect. Due to space constraint, this review focuses only on the relationship between adult-neurogenesis and affective disorders (see Box 2).

Overall, adult neurogenesis appears to be related both with cognitive and affective functions by plugging new neurons with unique functions into host mature circuits. Therefore, hippocampal circuitry is constantly remodeled by integrating maturing neurons, thereby altering the function of existing networks. Number of studies have revealed the functional uniqueness of this extreme form of cellular plasticity. Maturing adult-born granule cells receive first inhibitory inputs from local GABAergic interneurons and then form transient synapses onto pre-existing neighbor granule cells [69]. Once mature, the adult-formed granule cells enter into synaptic competition with the pre-existing ones, a competition that concerns both synaptic inputs from the entorhinal cortex and synaptic outputs to the CA3 and CA1 targeted regions [70, 71]. Because the newly-formed neurons are hyperexcitable, adult neurogenesis can lead to the formation of new and plastic synaptic connections, a feature that boosts cognitive functions such as pattern separation (the process by which overlapping or similar inputs are transformed into less similar outputs) and pattern completion (the reconstruction of complete stored representations from partial inputs) [72]. Adult-generated granule cells form connections with hippocampal interneurons that strongly inhibit neighboring pre-existing granule cells through a process called lateral inhibition. When new granule cells impinge onto interneurons located distantly in CA3 or CA1 regions, they provide feedforward inhibition [73]. Therefore, adding granule cells by the means of adult neurogenesis does not imply an overall excitation, on the contrary. Because new neurons provide both lateral and feedforward inhibition, adult neurogenesis contributes to the overall sparsification of population firing across the hippocampus [74]. Finally, in addition to the local action within the hippocampus, new neurons could be preferentially targeted by afferents originating from many brain regions. In that case, adult-born neurons appear as powerful hub capable of relaying the action of afferent pathways into the hippocampus [75].

Box 2 What do we mean by affective disorders? First coined by the French philosopher René Descartes (1596-1650), the term “affect” refers to the subjective experience of emotion or feeling. Its derivative “affective disorders” became rather ambiguous in the psychiatric field when it refers to disturbances of the emotional state seen across several pathologies. In its most common definition, affective disorders are superimposed on mood disorders (including major depressive disorder (MDD) and bipolar disorder (BD)). On the other hand, in a broader acceptance not retained here, affective disorders include mood disorders and anxiety disorders (generalized anxiety disorder, panic disorder, phobias), trauma and stress-related disorders (post-traumatic stress disorder, acute stress disorder) and obsessive-compulsive disorders.

## Body-brain interactions, adult neurogenesis, and affective states

**Fig. 2:**
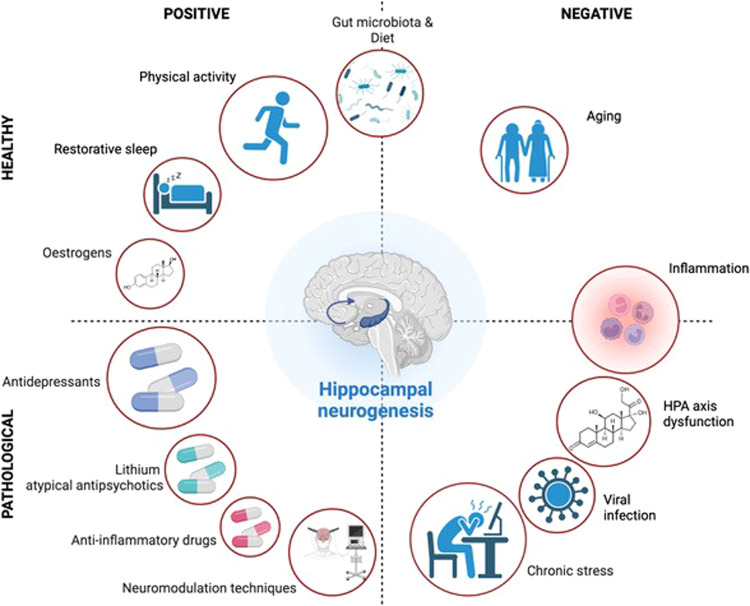
Factors potentially influencing hippocampal neurogenesis in healthy and pathological conditions. Numerous internal and external factors have been identified as potential regulators of hippocampal neurogenesis, either positively by stimulating neurogenesis (left side) or negatively by inhibiting it (right side). These factors may be present either in healthy conditions (upper part) or in pathological conditions (lower part). Created with BioRender.com.

As it is often the case in living organisms, important functions are subjected to multiple controls exerted by numerous regulators. Adult neurogenesis is no exception to that rule since it is regulated by a panel of endogenous brain-derived factors such as neurotransmitters and trophic factors. Moreover, several exogenous factors to the brain also influence adult neurogenesis (Fig. 2). The first factor to be studied extensively was the effect of stress and glucocorticoids on hippocampal neurogenesis and affective states [76]. Number of studies reported that repeated stress situations (social defeat, exposure to predator odor, restraint, pain conditioning, unpredictable chronic moderate stress), sleep deprivation and viral infection reduces hippocampal neurogenesis in rodents while caloric restriction, antidepressants or physical exercise increase it [77, 78].

**Fig. 3:**
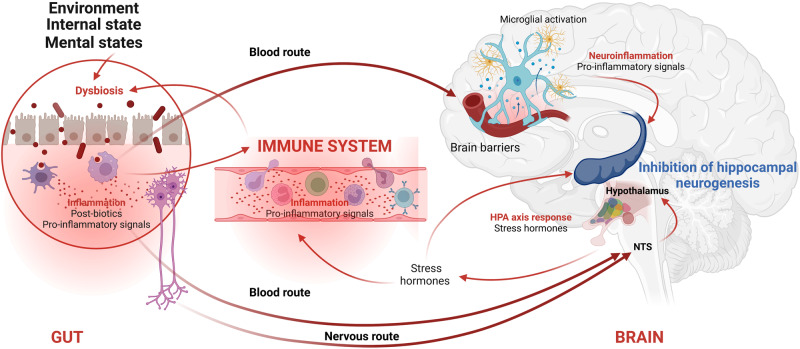
Gut, brain and immune system interactions. The environment, which encompasses all the external factors to which the individual is exposed (stress, diet, infection,…), can lead to a change in the intestinal microbiota. Stimulation of the immune system by post-biotics and cytokines produced by the intestinal immune system will send signals to the brain, by at least two routes: the bloodstream and the nervous route. The blood signals arriving at the brain’s barriers will cause activation of the barriers’ immune system that pass to the brain tissue, resulting in a neuroinflammatory state. The inflammatory signals are then relayed within the brain tissue by microglial cells (yellow). In hippocampus, inflammatory signals have an inhibitory effect on neurogenesis. In areas where the barriers are absent (e.g., in the circumventricular organs), the signals can be received directly in the brain and activate specific neuronal circuits. For example, an activation loop involves the nucleus tractus solitarii (NTS) and the hypothalamus, resulting in the secretion of stress hormones such as corticosterone [179] by hypothalamic–pituitary–adrenal (HPA) axis. Stress hormones have a deleterious effect on hippocampal neurogenesis. The direct nervous route involves the vagus nerve activity that transmits signals directly to the NTS. Created with BioRender.com.

More recently, the trillions of microorganisms which reside in our gastrointestinal tract have been also reported to profoundly influence adult neurogenesis. This has shed new light on the processes linking environmental factors, stress, adult neurogenesis, and affective states. Because the composition of the gut microbiome is influenced by several lifestyle factors such as birth mode (vaginal *vs*. cesarean section), diet composition, physical exercise, stress or aging [79], it is not clear whether lifestyle factors act directly on the process of hippocampal neurogenesis, or indirectly by changing the microbiome-derived neurogenic factors (Fig. 3), or both. In any case, mounting evidence through germ-free (GF) rodents [80], microbiota transplant studies [81, 82], and microbiota-targeted interventions such as those mediated by dietary supplementation or antibiotics treatment [83, 84] highlight the importance of the gut microbiota composition for adult hippocampal neurogenesis (reviewed in [85]). For instance, the mere transfer of the microbiota from stressed mice to naive mice is sufficient to transfer the hallmarks of depression, including decreased hippocampal neurogenesis, altered serum metabolite levels, and expression of despair-like behaviors [86, 87].

Patients suffering from MDD have been found to harbor unique signatures in their gut microbiomes. Both microbial diversity and microbial richness are significantly decreased (a process called dysbiosis) in depressed patients, and when this atypical microbiota signature is transferred to healthy, naive rats, the recipient rodents showed anxiety-like and anhedonia behaviors [88]. To achieve these effects, the gut microbiota requires either the bloodstream [86, 87] or a neural route through the vagus nerve [89] (Fig. 3). Remarkably, even the efficacy of an antidepressant such as fluoxetine, is sensitive to changes in gut microbiota composition. For instance, stress-induced changes in gut microbiota dampen fluoxetine efficacy via depletion of brain serotonin [87]. Hence, the anti-neurogenic microbiome exerts its inhibitory effect by switching tryptophan catabolism from serotonin synthesis to the kynurenine production.

Despite all the recently acquired knowledge, further investigations are needed to better understand how the gut microbiome could mechanistically impact the hippocampal neurogenesis before considering a potential microbiota-based treatment for affective disorders. Today, the clinical outcomes about the ability of the gut microbiota to boost human adult hippocampal neurogenesis are rather limited. The identification of clinical biomarkers of microbiota dysfunction associated with affective disorders is a key step to consider novel therapeutic options to treat mental disorders.

## Inflammation: the missing link between environment and adult neurogenesis?

One of the main candidates that strongly connects microbiota dysbiosis to the degree of adult neurogenesis are immune factors driving neuroinflammation (Fig. 3). We define here neuroinflammation as a set of cerebral nonspecific immune responses triggered by various disturbances to the homeostatic state of the organism. These responses are mainly coordinated by innate or adaptive immune factors that particularly target the nervous system. Under physiological conditions, brain immunocompetent cells such as microglia, or their derivate molecular signaling, can boost hippocampal neurogenesis [90]. In contrast, other immune challenges can reduce adult neurogenesis and induce depressive-like “sickness behavior” in both animals and humans [91]). For instance, mounting evidence shows that LPS (lipopolysaccharides), which elicits depressive-like behaviors as well as neuroinflammation, attenuates adult hippocampal neurogenesis in rodents [92] together with a decrease in (Brain-derived neurotrophic factor) expression [93]. Chronic LPS reduces the dendritic length and postsynaptic cluster density of immature newborn granule cells in the hippocampus in mice [94]. These changes are also observed after IFN-α injection, which alters hippocampal cell proliferation, survival, and neuronal differentiation in both rats and mice (reviewed in ref. [95]).

In line with this, previous reports have described the existence of a strong association between depression and peripheral markers of inflammation, in both blood and cerebrospinal fluid [96]. These results were confirmed by more recent cumulative meta-analysis showing interleukin-6 and C-reactive protein (CRP) to be most strongly associated factors with depression [97]. These analyses also show that a significant number of cytokines (including IL1α, IL1β, TNFα, IL2, IL12, IL18) and receptors (sIL-1RA, sIL-6R) are abnormally high in patients suffering from depression [98] and that these disturbances are present in the acute and stability phases, in MDD and BD [99].

Pro-inflammatory cytokines are known to influence serotonin metabolism by promoting the functioning of the indoleamine 2,3-dioxygenase (IDO), thus diverting tryptophan metabolism towards the kynurenine pathway instead of the serotonin synthesis pathway. Activation of kynurenine pathway leads to the production of quinolinic acid which is an agonist of glutamatergic NMDA receptor. Thus the switch to kynurenine pathway may lead to excitotoxicity and HPA axis dysregulation, two conditions that alter hippocampal neurogenesis (reviewed in ref. [95]). Not surprisingly, the kynurenine/tryptophan ratio (an indicator of the activation of the first step of the kynurenine pathway) is increased in the plasma and CSF of patients having received IFN-α and correlated with depressive symptoms [100, 101]. An imbalance in favor of the kynurenine pathway associated with a decrease in serotonin, is also correlated with depression scores in patients with mastocytosis [102]. Thus, the initial serotonergic hypothesis of depression (reviewed in ref. [103]) is consistent with an involvement of serotonin-kynurenine-inflammatory pathway, and supports the more general concept of “inflammatory hypothesis” in the pathophysiology of depression [104]. Indeed, adult hippocampal neurogenesis might be one of the most important processes that bridges neuroinflammation to affective disorders, although it remains to be established whether neuroinflammation is a consequence or a key etiological factor causing depression.

Further support to the neuroinflammation theory of affective disorders involving changes in the adult neurogenesis, originate from studies of neuroglial cells. Microglial cells migrate and become activated during cytokine-induced neuroinflammation (see for instance [105]) and their activity has been associated with impaired hippocampal neurogenesis and depressive states [106]. Impairment of neurogenesis by microglial activation might be responsible for triggering, or potentiating, inflammatory-associated depressive symptoms, as found in animal models of depression (reviewed in ref. [107]). Manipulating the microglial phenotype is a potential strategy to treat affective disorders. Some translational data have shown that the action of antidepressant molecules such as ketamine may involve direct modulation of the activation state of microglial cells [108].

## It takes two to tango: Affective disorder treatments and adult neurogenesis

Data obtained in rodents have shown that conventional antidepressants classes, such as selective serotonin reuptake inhibitors, noradrenaline reuptake inhibitors, tricyclics or monoamine oxidase inhibitors, all increase hippocampal neurogenesis [109, 110], or prevent their reduction by chronic stress exposure [111–113]. The two to four weeks delay in action observed for these treatments agree with a neurogenesis-dependent mechanism. However, it is important to note that these conventional antidepressant drugs also exhibit other effects such as dendritic spine density regulation in animal models [114, 115]. Interestingly, it has recently been shown that fluoxetine can stimulate progenitor renewal in peripheral organs such as bone marrow [116], adding more complexity to the action of antidepressants on the brain and peripheral organs.

The latest treatments developed, or under development, in the field of depression, (i.e., ketamine, esketamine or psilocybin), have a clear effect on neuroplasticity, with a rapid effect on BDNF production and dendritic spine regrowth. This mode of action explains their rapid effectiveness although repeated administrations are required to produce long-lasting antidepressant effect (for ketamine see [117–119]; for psilocybin [120, 121]). Importantly, it has been shown that both ketamine and psilocybin also have effect on hippocampal neurogenesis [122, 123]. Finally, it has been shown that neuromodulation treatments (i.e., therapeutic methods that modulate neural circuitries activity by electromagnetic stimuli, electric current or ultrasound) have a clear effect in stimulating neurogenesis in rodents (see for review [124]). Robust data pointed out the effect of electroconvulsive therapy (ECT) on hippocampal neurogenesis [125, 126]. Other stimulation modalities such as repetitive transcranial magnetic stimulation [127], deep brain stimulation [128] and vagus nerve stimulation [129] also alter adult neurogenesis [124].

The link between treatments and neurogenesis is supported by post-mortem studies showing that patients exposed to antidepressant treatments exhibit increased hippocampal neurogenesis compared to untreated depressed patients [130–132] (see Table 1). Clinical studies have also shown that hippocampal volume decrease reported in MDD patients was counteracted by antidepressant drugs [133, 134]. In the same line, it was shown that hippocampal volume loss is a predictor of antidepressant response in patients [135, 136] or in patients treated with ECT [137].

Although low-grade inflammation has been evidenced in the depressed patient population and anti-inflammatory drugs showed encouraging results in pilot studies, anti-inflammatory drugs are not indicated as monotherapy or add-on treatment for depression yet (see for review [138]). However, in mice subjected to chronic stress, minocycline can counteract anxio-depressive-like phenotype and stress-induced microglial activation associated with decrease in hippocampal neurogenesis [139].

Concerning BD, the mood stabilizer lithium (Li) is the gold standard treatment, but other molecules such as antipsychotics (e.g., clozapine or haloperidol) and anticonvulsants (e.g., lamotrigine or valproic acid), could be used. In mice, several studies suggest that Li could be acting though regulation of neurogenesis, although clear direct evidence is still missing [140, 141]. Recent in vitro experiments showed that high doses of Li treatment in human hippocampal progenitors increase the generation of neuroblasts [142]. In humans, MRI data demonstrated that Li accumulates in the hippocampus of BD patients [143] and numerous studies have shown an increase in hippocampal volume after Li treatment (see for review [144]). Changes in hippocampal volume correlate with response to Li treatment and cognitive functioning improvement [145, 146].

Anticonvulsant mood stabilizers in particular valproic acid—which was widely used as a mood stabilizer before teratogenic effects and increased risk of neurodevelopmental disorders when prescribed to pregnant women were discovered - are common mood stabilizers [147, 148]. Valproic acid stimulates hippocampal neurogenesis and neuronal growth in rodent cortex [149]. In BD patients, valproic acid was not associated with modification in hippocampus volume contrary to Li treatment [145, 150]. Data are much more limited for lamotrigine, a treatment especially used for bipolar depression prophylaxis. Inconsistent results exist showing both increase [151] or reduction in neurogenesis rat hippocampus [152].

Finally, antipsychotic treatments can also be used as mood stabilizers. Antipsychotic drugs are divided into typical or first-generation antipsychotics (such as haloperidol and risperidone), characterized by dopamine D2 receptor antagonism and atypical (such as aripiprazole, olanzapine and clozapine) that share D2 receptor antagonism and additional pharmacological properties, including actions on serotonergic transmission. Chronic administration of atypical antipsychotics including olanzapine [153, 154], aripiprazole, and clozapine [154] were reported to increase hippocampal neurogenesis, whereas haloperidol has no effect [155–157] or a negative one [154]. Interestingly, some studies found a correlation between antipsychotics treatment and increase in neurogenesis in other brain areas including striatum, prefrontal cortex, and SVZ [155, 157]. The positive effect of atypical antipsychotics on neurogenesis may result from partial 5HT2A receptor antagonism and/or 5HT1A receptor agonism affecting the GSK3β/β-catenin pathway and BDNF production [158]. Therefore, the classification between typical and atypical antipsychotics could be based on their effect on neurogenesis rather than their pharmacological properties regarding neurotransmitter receptors affinity. Interestingly, these molecules are also used in the treatment of schizophrenia spectrum disorder patients, in which a decrease in hippocampal volume has also been demonstrated [159] and reversed by aripiprazole treatment [160].

Finally, other non-pharmacological interventions with a link to neurogenesis are discussed in the management of mood disorders, such as physical activity (see for review [161]). In mice, voluntary physical exercise is associated with an increase in hippocampal neurogenesis [162–164] and with enhancement of cognitive functions [165–167]. Physical exercise’s effect on adult neurogenesis relies on circulating blood factors. Thus, transfer of plasma from aged exercised mice to aged sedentary mice has a positive effect on neurogenesis and cognitive functioning [168].

To conclude, the mechanism of action of mood disorder therapeutic drugs, mostly defined by their receptor affinities, raises important issues. Bearing in mind the ensemble of studies, molecules and treatments used to handle affective disorders might be considered through the prism of adult neurogenesis.

## Modeling affective disorders or modeling affective functions?

The current limitation of translational approach in affective disorders lies in the heterogeneity of these diseases based on the Diagnostic and Statistical Manual of mental disorders (DSM, Diagnostic and statistical manual of mental disorders (5th ed., text rev),. American Psychiatric Association, 2022). The syndromic definition of diseases by a combination of symptoms that may vary from one patient to another, without associated pathophysiology or biomarkers, groups under the same entity diseases that are most likely different. This is a major obstacle to modeling. To solve this conundrum, these diagnoses should be dismantled into simpler, or even unsupervised entities, where specific symptoms will be associated with a known pathophysiology.

To enable this paradigm shift, it is essential to accurately describe the dimensions or functions affected in depression or mania. From this perspective, the approach based on the Research Domain Criteria framework was proposed to group clinical symptoms in six domains and facilitate research on mood disorders [169]. However, the major limitation in defining these domains is the absence of a link with a pathophysiological mechanism or biomarker. Thus, to define clusters of symptoms related to a given pathophysiology, it is necessary to return to specific symptoms or functions.

**Fig. 4:**
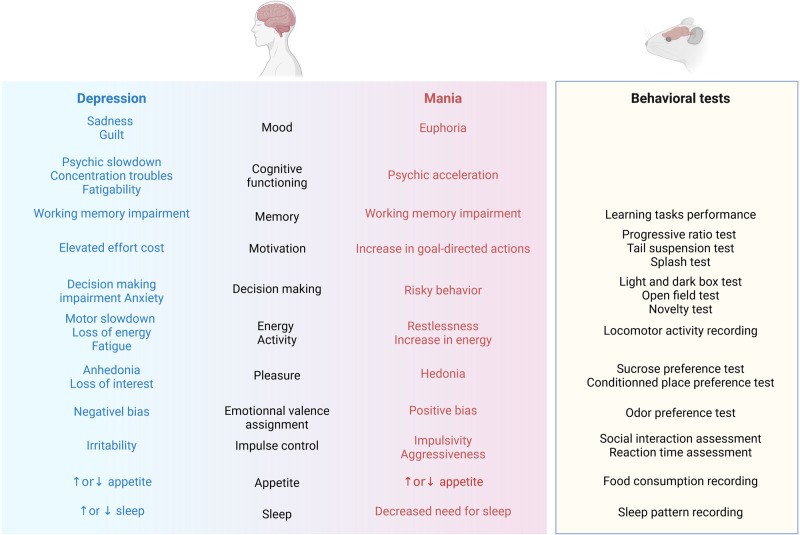
Assessment of affective functions linked to clinical symptoms of mania or depression. Mood disorders, characterized by depressive state (blue) and maniac state (red), can be described by impairment of affective functions (center). These functions should be tested with specific behavioral analysis in animal models (yellow box). Created with BioRender.com.

In patients, disturbances of several functions have been described: mood itself, cognitive functioning, memory, motivation, decision making, energy and activity, pleasure, emotional valence assignment, impulse control, appetite, and sleep. Most of these dimensions can be tested in animals with the batteries of behavioral tests available [170] (Fig. 4). Some dimensions, such as assessment of emotional valence shift by odor preference test, are newly proposed in the field [171]. It is important to note that some dimensions described by patients remain impossible to translate into animal models: mood itself with feelings of sadness, guilt, pessimism, or on the contrary euphoria or irritability; complex cognitive functions such as planning capabilities or suicidal ideation. Moreover, accessible behavioral tests should be considered with caution and need substantial refinements, because the readouts were obtained from short, highly controlled tests in impoverished environments and social contexts, quite distinct from the complexity of human behaviors [172].

Currently, animal models of mood disorders are mainly based on environmental, pharmacological and genetic manipulations (for review see [170, 173]) and seek to reproduce the symptoms seen in humans. For BD, the cyclicity of the symptoms is not reproducible in rodents and separate models are used for depressive phases and manic phases. Some of these models are based on well-known risk factors for mood disorders, such as repeated stress situations or HPA axis dysfunction for depression [170]. However, caution must be taken regarding the possible extension of specific models. For example, a depressive state can be induced by early maternal separation in rodents, which is rather related to early childhood trauma and is a risk factor for BD or other psychiatric disorders. Some models are only developed in male mice while the depressed patient population is predominantly women (Box 3). Another source of confusion consists in mixing models of acute stress with models of chronic manipulations leading to long-lasting anxio-depressive-like phenotypes. For instance, acute immunological stress such as unique LPS administration induces behavioral disturbances - called sickness behavior - that are similar to those seen in depression, yet they are only transient [174]. Thus, caution must be taken regarding the possible extension of this model to the inflammatory disturbances seen in depression, which correspond to chronic low-grade inflammation. For instance, induction of sepsis, which corresponds to an extreme form of acute inflammatory stress with severe sickness behavior, induces long-term anxiety symptoms suggestive of post-traumatic stress disorder rather than depressive disorders [175].

After checking the translational validity of the selected model, the specific functions affected could be described. Hence, some models reproduce only one dimension of the pathology concerned and others could copy negative emotional bias or anhedonia, as a landmark of depressive states. This approach could be more of a strength than a limitation, allowing to link these functions, or clusters of functions, with pathophysiological mechanisms. It is also likely that animals subjected to different models of depression (chronic stress, chronic exposure to corticosterone, chronic exposure to an inflammatory stimulus…) present different anxiety-depressive patterns when assessed by the same battery of behavioral tests. Thus, the question to be addressed could no longer be whether an animal or a human is depressed, but rather in what way is this animal or human depressed?

Box 3 Does sex matter? The systematic use of mice of both sexes to model mood disorders has been poorly addressed, although sex differences get more attention than in the past (for review see [185]). For instance, a large proportion of experiments have been carried out on male mice when two-thirds of patients are women [186]. The vulnerability of women to depression could be linked to a particular mechanism of regulation of neurogenesis. For instance, estrogen stimulates hippocampal neurogenesis (reviewed in [187]), by increasing the rate of proliferation [188] while male hormones increase the survival of new neurons but not cell proliferation [189].

## Concluding remarks

This review aims to synthesized current evidence of the production of new neurons in the adult brain, including humans, its regulation from both external and internal stimuli, and its relationship with affective disorders. While there are several studies showing that reduced hippocampal neurogenesis is associated with depression and anxiety, the causal link between the two is still a topic of intense research. Animal studies provide strong support for a causal link between decreased hippocampal neurogenesis and depression-like behaviors. For instance, disrupting hippocampal neurogenesis induces depression-like behaviors in rodents, while increasing neurogenesis has an antidepressant-like effect. Similarly, antidepressant drugs stimulate neurogenesis and alleviate depression-like behaviors. However, these findings have not been consistently replicated, and as described above some studies have failed to find a direct causal link between adult neurogenesis and affective disorders. Furthermore, it is not clear whether changes in adult neurogenesis are a cause or a consequence of affective disorders, or whether other factors such as chronic stress or inflammation may be involved. Therefore, while the evidence suggests that adult neurogenesis may play a role in affective disorders, more research is needed to fully understand the causal relationship between the two. Demonstrating the role of adult neurogenesis in human affective disorders is a challenging task because it is not possible to manipulate neurogenesis in the same way as in animal models. The study of adult neurogenesis is also puzzling and tedious because brain imaging techniques have limitations when it comes to resolving this process, although some tentative have been tried in the past [176]. There are several approaches we foresee to characterize the relationship between adult neurogenesis and affective disorders, in humans:

1. Neuroimaging studies: We believe that in vivo imaging approaches such as functional magnetic resonance imaging (fMRI) and positron emission tomography (PET) can be used to measure changes in brain cells activity (neurons but also microglial cells with specific tracers) and neurochemistry associated with depression. These techniques provide information about the functional connectivity and activity of the hippocampus and may indirectly suggest changes in adult neurogenesis. When applied to patients, SPECT (single photon emission computed tomography) and PET should be helpful in linking changes occurring in behavior with brain structural/functional changes.
2. Post-mortem studies: Analysis of brain tissue from individuals who have died with a history of depression could help to establish whether there are structural differences in the hippocampus, as well as in other brain regions, compared to individuals without a history of mood disorders. The recent new markers and molecular signatures of adult human neurogenesis should help in this quest.
3. Pharmacological treatments: New drugs such as psychedelics should reveal new actions of antidepressant treatments on adult neurogenesis in humans.
4. Longitudinal studies: Studies that measure changes in adult neurogenesis over time in individuals with mood disorders should strengthen the field. By comparing changes in adult neurogenesis in individuals who respond to treatment *versus* those who do not, clinicians could gain insights into the role of adult neurogenesis in the development and treatment of affective disorders.
5. Understanding the mechanisms that regulate adult neurogenesis. In addition to these promising new methodological strategies, it may be useful to investigate the underlying molecular and cellular mechanisms that link neurogenesis to affective disorders. In this line, studying the role of gut microbiota, stress, inflammation, and neurotrophic signaling pathways, should be enlightening.
6. Use of human in vitro models: In vitro models of adult neurogenesis can be used to investigate how neurogenesis is affected in brain disorders [177, 178]. One common approach is to culture primary neural cells. Treating them with a serum of patients, the proliferation, differentiation, and maturation of neural stem cells and progenitor cells can be studied. Also, human/patient induced Pluripotent Stem Cells (iPSCs) can be used to generate human neurons, providing a platform to study the entire process of adult neurogenesis. Finally, 3D brain organoids are three-dimensional cultures of human brain cells that mimic certain aspects of brain development. They can be used to model aspects of adult neurogenesis within a more complex tissue structure.

Overall, a multi-disciplinary approach that combines clinical, neuroimaging, molecular, and pharmacological methods will be necessary to fully elucidate the complex relationship between adult neurogenesis and affective disorders, and to develop effective treatments for these conditions. In the meantime, it is interesting to realize how much the existence of adult neurogenesis in humans is becoming a dogma after several decades of heated debate.
